# Malnutrition mediates the association between handgrip status and asthma risk: an observational and prospective cohort study from multiple European countries

**DOI:** 10.3389/fnut.2025.1555888

**Published:** 2025-07-07

**Authors:** Jun Wen, Xiaowen Shi, Yan Liu, Rongjuan Zhuang, Shuliang Guo, Jing Chi

**Affiliations:** Department of Respiratory and Critical Care Medicine, The First Affiliated Hospital of Chongqing Medical University, Chongqing Medical University, Chongqing, China

**Keywords:** handgrip strength (HGS), relative handgrip strength (RHGS), asthma, malnutrition, machine learning, Shapley Additive Explanations (SHAP)

## Abstract

**Background:**

Epidemiological investigations on the association of handgrip status and asthma risk still remain understudied. This research aims to investigate the associations of handgrip strength (HGS), relative handgrip strength (RHGS), low HGS, and asthma risk, as well as the mediating role of nutritional status, using data from the Survey of Health, Ageing and Retirement in Europe (SHARE).

**Method:**

This investigation included 27,185 participants for a cross-sectional study and 18,047 participants for a prospective cohort study from SHARE. Four machine learning models, the Shapley Additive Explanations (SHAP) model, restricted cubic spline (RCS), cumulative occurrence curve, logistic regression, and Cox regression were used to comprehensively evaluate the performance of handgrip status in predicting asthma risk. Finally, the mediation effect model was employed to evaluate the role of nutritional status in the relationship between grip strength and asthma risk.

**Result:**

The cross-sectional investigation suggested that both HGS (OR: 0.98, 95% CI: 0.98–0.99) and RHGS (OR: 0.61, 95% CI: 0.51–0.73) were negatively linked to the risk of asthma, and low HGS was a risk factor for asthma (OR: 1.52, 95% CI: 1.24–1.87). And the prospective cohort investigation with a median follow-up time of 30 months further confirmed that both HGS (HR: 0.98, 95% CI: 0.97–1.00) and RHGS (HR: 0.52, 95% CI: 0.37–0.73) were negatively linked to the risk of asthma. Among the four machine learning models used to evaluate handgrip status and the risk of asthma, eXtreme Gradient Boosting (XGBoost) showed better predictive performance. The SHAP model based on XGBoost suggested that the top five crucial indicators for predicting asthma risk were RHGS, HGS, country, age, and chronic lung disease. Finally, the mediation effect model suggested that malnutrition partially mediated the relationship between low HGS and increased risk of asthma, with a mediation proportion of 2.71%.

**Conclusion:**

This investigation suggested that lower HGS and RHGS were linked to a higher risk of asthma, and handgrip status could be used as an independent marker of asthma risk in European populations. And malnutrition partially mediated the relationship between low HGS and asthma risk. Improving muscle strength could be a potential preventive strategy against asthma, with implications for public health and clinical practice.

## 1 Introduction

Asthma is a common respiratory disease characterized by tracheal remodeling, and persistent airflow limitation (1). In 2023, Global Initiative for Asthma (GINA) defined asthma as a serious global health problem, with more than 360 million people suffering from asthma worldwide and about 1,000 people dying from asthma every day (2). Although its global impact is widely acknowledged, the burden of asthma in Europe is particularly substantial. Asthma affects an estimated 30 million children and adults under 45 years old across Europe, with national prevalence rates ranging from 5 to 11% (3). Asthma has a variety of symptoms, and severe cases have frequent onset and long course of disease, which is a huge pressure on the patient’s physiology and psychology and seriously affects the patient’s life (4). Although the overall mortality rate of asthma is decreasing year by year, the social and personal financial pressure caused by long-term treatment persist (5). These unfavorable factors show that asthma remains a significant social and public issue, requiring continued vigilance and concerted efforts for effective management and control.

Handgrip strength (HGS) is a widely used and convenient measure of muscle strength in clinical and research settings, owing to its simplicity, non-invasiveness, and low cost, and has been widely used to assess overall muscle strength and fitness (6, 7). Some scholars also believe that relative handgrip strength (RHGS) is more accurate for measuring muscle strength due to individual differences in HGS and a high correlation with age, gender, height, weight, and other factors (8, 9). The combination of the two can provide clinical tools for assessing nutritional status and health risks across the lifespan from adolescence to old age (6, 10). In addition, grip strength has been shown to be associated with all-cause mortality and disease prognosis (11, 12). At the same time, grip strength, as an indicator of muscle strength, reflects the degree of health and has been linked to various conditions, including cardiovascular disease, neurological disease, cancer, and so forth (13–15).

In the respiratory system, grip strength is associated with late quality of life in chronic obstructive pulmonary disease (COPD), clinical outcomes in moderate to severe asthma, and dilated nutritional status in patients with non-cystic fibrosis bronchiectasis (16–18). Recent studies have further emphasized the relationship between muscle strength and nutritional status in patients with respiratory diseases, suggesting that malnutrition and muscle weakness may exacerbate disease progression and hinder recovery in COPD and asthma patients (18–20). For instance, reduced HGS measured during hospitalization can serve as a valuable predictor of both in-hospital and post-discharge acute exacerbation risks in COPD patients (21). Moreover, recent research has suggested that improving muscle strength through nutritional interventions may enhance clinical outcomes and reduce exacerbation rates in COPD patients (22, 23). Additionally, previous studies have indicated that grip strength could be used as a diagnostic tool for assessing frailty risk in elderly asthma patients (24). Hesselberg et al. (25) has confirmed a significant link between grip strength and lung function indicators.

Epidemiological investigations on the association between handgrip status (RHGS, HGS, low HGS) and asthma risk still remain understudied. This research aims to investigate the association between handgrip status and asthma, as well as the mediating role of nutritional status, using cross-sectional and prospective cohort studies and data from the SHARE database.

## 2 Methods

### 2.1 Data source and study design

SHARE is biannual and longitudinal investigation encompassing various European nations, focusing on adults aged 50 years and above (26). The data collection involved computer-assisted personal interviews covering multiple areas, including demographics, socioeconomic characteristics, living situations, and both physical and mental health graphics. This investigation included 27,185 participants for a cross-sectional study and 18,047 participants for a prospective cohort study from the SHARE wave 1 to 2. Screening of cross-sectional study population: (1) people without asthma (*n* = 13,710); (2) people with missing HGS or BMI (*n* = 2,811); (3) people with missing covariates (*n* = 255). The prospective cohort study population was screened as follows: (1) individuals with asthma or missing data (*n* = 15,092); (2) individuals with missing HGS or BMI or time (*n* = 10,405); and (3) individuals with missing covariates (*n* = 417). Supplementary Figure S1 shows the specific screening process of the population analyzed in this investigation. Supplementary material provides more details on the study populations.

### 2.2 Measurement of RHGS, HGS, and low HGS

The handheld dynamometer (Smedley, S Dynamometer, TTM, Tokyo, 100 kg) was employed to measure each HGS. Participants were instructed to maintain a neutral wrist position and keep the upper arm vertical against the trunk, either standing or seated, with the elbow flexed at a 90° angle. This was in accordance with the SHARE protocol. The survey was conducted in the residences of the participants. Measures were executed. The HGS measurement was administered by the same trained interviewers who conducted the in-person home computer-assisted personal interviews. They verbally encouraged participants to squeeze the dynamometer with maximum effort for a few seconds, according to standardized instructions. HGS in this investigation was defined as the highest value of either hand. *RHGS (m^2^) = HGS (kg)/BMI (kg/m^2^)*. Low HGS was defined as a maximal HGS of less than 27 kg for males or less than 16 kg for females (27).

### 2.3 Covariates

The covariates in this investigation were derived from SHARE, and they included age, country (Austria, Germany, Sweden, Netherlands, Spain, Italy, France, Denmark, Greece, Switzerland, Belgium, Israel), gender, education (low, medium, and high education), drinking status (defined as consuming more than 2 glasses of alcohol almost every day), smoking status, malnutrition (BMI less than 18.5 was defined as malnutrition), hypertension, diabetes, chronic lung disease, heart attack, stroke, arthritis, and cancer status. The median follow-up time for the cohort study was 30 months.

### 2.4 Statistical analysis

For categorical data, the *p*-value was ascertained using the chi-square test. The Kruskal-Wallis rank-sum test was implemented to compute the p-value for continuous variables. For continuous variables that lacked a normal distribution, this research implemented the median and IQR. Categorical variables were described using proportions. Initially, this investigation employed three logistic regressions (for cross-sectional populations) and Cox proportional hazards regression analyses (based on longitudinal populations) to examine the relationship between handgrip status (RHGS, HGS, low HGS) and asthma risk. Trend tests and restrictive cubic splines (RCS), based on models that adjust for all covariates (age, country, gender, education, drinking status, smoking status, hypertension, diabetes, chronic lung disease, heart attack, stroke, arthritis, and cancer status), can further quantify the relationship between handgrip status and asthma risk. Support vector machines (SVM), random forests (RF), decision trees (DT), and XGBoost were chosen to construct the model for the purpose of comparing the efficacy of various machine learning models in predicting asthma risk. The area under the curve (AUC) acted as the crucial performance evaluation metric to ascertain the best machine learning model to assess the performance of handgrip status in predicting asthma risk. Then, this investigation applied the SHAP based on XGBoost with better predictive performance to assess the importance of each variable in forecasting asthma risk and to explain the relationship between each variable and asthma risk. Afterwards, the cumulative risk curve was used to evaluate the relationship between handgrip status with the occurrence of asthma. This investigation also applied the area plots and matrix plots, based on the “contsurvplot” package (28), to evaluate the effect of grip strength on the absence of asthma during the follow-up period. Subsequently, this investigation implemented the mediation effect model to assess the contribution of nutritional status to the association between low grip strength and asthma risk. Missing covariates included age, drinking status, education, marriage, and smoking status. The proportion of each missing covariate was less than 1%, and the missing covariates in this study were addressed through multiple imputations (“mice” package). Conduct sensitivity analyses of the primary findings using the data generated through multiple imputations. Every statistical analysis was carried out with R 4.4.1. Statistical significance was defined as a *p*-value of less than 0.05. The Supplementary material contain more detailed information about statistical analysis.

## 3 Results

### 3.1 Baseline characteristic

Tables 1, 2 summarize basic characteristics of the investigated populations. The cross-sectional investigation (Table 1) encompassed 27,185 individuals of a mean age of 63.30 years, with 1,194 receiving a diagnosis of asthma. Some variables, including age, country, education, marriage, smoking, heart attack, hypertension, chronic lung disease, arthritis, and malnutrition, exhibited statistically significant variations among populations with and without asthma. The longitudinal investigation (Table 2) encompassed 18,047 individuals with a mean age of 63.09 years, of whom 329 had newly diagnosed asthma. Some variables, including gender, age, country, education, marriage, heart attack, hypertension, diabetes, chronic lung disease, heart attack, stroke, and arthritis, exhibited statistically significant variations among populations with and without asthma. Individuals with asthma were more likely to experience low HGS and had lower RHGS and HGS levels.

**Table 1:** Baseline characteristics of study population based on the cross-sectional investigation.

Variable	Non-asthma (*N* = 25,991)	Asthma (*N* = 1,194)	*p*-value
Gender (%)			0.132
Female	54.40%	56.62%	
Male	45.60%	43.38%	
Age (years)	62.00 (55.00–70.00)	63.00 (56.00–72.00)	0.007
Country (%)			<0.001
Austria	4.91%	4.44%	
Germany	10.13%	6.95%	
Sweden	9.88%	17.17%	
Netherlands	9.98%	9.38%	
Spain	7.40%	6.37%	
Italy	8.32%	8.79%	
France	9.94%	10.39%	
Denmark	5.65%	11.47%	
Greece	9.55%	6.20%	
Switzerland	3.37%	2.51%	
Belgium	13.30%	8.79%	
Israel	7.56%	7.54%	
Education (%)			<0.001
Low education	49.86%	55.44%	
Medium education	30.57%	26.97%	
High education	19.57%	17.59%	
Marriage (%)			0.004
Single	24.38%	27.55%	
Married	74.01%	70.10%	
Other	1.61%	2.35%	
Smoking status (%)			<0.001
Current smoking	19.96%	17.50%	
Past smoking	51.92%	46.24%	
Never smoking	28.12%	36.26%	
Drinking 2 glasses every day (%)			0.545
No	87.09%	87.69%	
Yes	12.91%	12.31%	
Hypertension (%)			<0.001
No	68.96%	62.48%	
Yes	31.04%	37.52%	
Diabetes (%)			0.092
No	90.42%	88.94%	
Yes	9.58%	11.06%	
Chronic lung disease (%)			<0.001
No	96.13%	77.30%	
Yes	3.87%	22.70%	
Heart attack (%)			<0.001
No	88.55%	81.74%	
Yes	11.45%	18.26%	
Stroke (%)			0.053
No	96.90%	95.90%	
Yes	3.10%	4.10%	
Arthritis (%)			<0.001
No	82.66%	71.61%	
Yes	17.34%	28.39%	
Cancer (%)			0.510
No	94.74%	94.30%	
Yes	5.26%	5.70%	
Low HGS (%)			<0.001
No	93.33%	88.36%	
Yes	6.67%	11.64%	
Malnutrition (%)			<0.001
No	98.94%	97.82%	
Yes	1.06%	2.18%	
RHGS (m^2^)	1.28 (0.96–1.66)	1.17 (0.84–1.57)	<0.001
HGS (kg)	32.00 (25.00–43.00)	30.00 (23.00–40.00)	<0.001

The median and IQR for continuous variables with a non-normal distribution. Proportions were utilized for describing categorical variables.

**Table 2:** Baseline characteristics of study population based on the longitudinal investigation.

Variable	Non-asthma (*N* = 17,718)	Asthma of new onset (*N* = 329)	*p*-value
Gender (%)			0.028
Female	54.99%	61.09%	
Male	45.01%	38.91%	
Age (years)	62.00 (55.00–70.00)	65.00 (57.00–73.00)	<0.001
Country (%)			<0.001
Austria	5.28%	6.38%	
Germany	8.07%	9.12%	
Sweden	9.06%	8.81%	
Netherlands	8.85%	10.33%	
Spain	7.05%	6.08%	
Italy	8.42%	11.85%	
France	9.54%	8.51%	
Denmark	6.12%	9.42%	
Greece	11.75%	7.29%	
Switzerland	3.60%	3.04%	
Belgium	14.56%	8.21%	
Israel	7.70%	10.94%	
Education (%)			<0.001
Low education	49.46%	60.49%	
Medium education	30.37%	25.53%	
High education	20.17%	13.98%	
Marriage (%)			<0.001
Single	24.84%	33.74%	
Married	75.16%	66.26%	
Smoking status (%)			0.889
Current smoking	19.58%	18.54%	
Past smoking	52.48%	52.89%	
Never smoking	27.94%	28.57%	
Drinking 2 glasses every day (%)			0.251
No	86.91%	89.06%	
Yes	13.09%	10.94%	
Hypertension (%)			0.017
No	68.77%	62.61%	
Yes	31.23%	37.39%	
Diabetes (%)			<0.001
No	90.79%	83.28%	
Yes	9.21%	16.72%	
Chronic lung disease (%)			<0.001
No	96.65%	73.56%	
Yes	3.35%	26.44%	
Heart attack (%)			<0.001
No	88.93%	80.24%	
Yes	11.07%	19.76%	
Stroke (%)			0.049
No	97.01%	95.14%	
Yes	2.99%	4.86%	
Arthritis (%)			<0.001
No	82.41%	72.04%	
Yes	17.59%	27.96%	
Cancer (%)			0.986
No	95.16%	95.14%	
Yes	4.84%	4.86%	
Low HGS (%)			<0.001
No	94.20%	89.36%	
Yes	5.80%	10.64%	
Malnutrition (%)			0.612
No	99.06%	98.78%	
Yes	0.94%	1.22%	
RHGS (m^2^)	33.00 (25.00–43.00)	30.00 (23.00–37.00)	<0.001
HGS (kg)	1.28 (0.97–1.66)	1.16 (0.80–1.40)	<0.001
Follow time (month)	30.00 (26.00–32.00)	30.00 (28.00–33.00)	0.002

The median and IQR for continuous variables with a non-normal distribution. Proportions were utilized for describing categorical variables.

### 3.2 Malnutrition mediates the association between handgrip status and asthma risk based on the cross-sectional investigation

**Figure 1:**
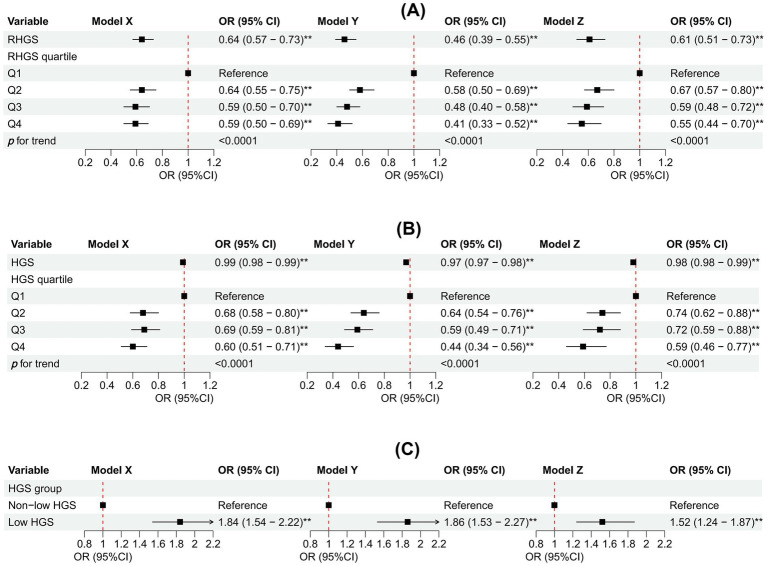
Association between RHGS **(A)**, HGS **(B)**, low HGS **(C)**, and asthma based on the cross-sectional investigation. Model X adjusted for none. Model Y adjusted for gender, country, and age. Model Z = Model Y + adjusted for education, marriage, malnutrition, smoke, drinking, heart attack, hypertension, stroke, diabetes, chronic lung disease, arthritis, cancer. Q1-Q4 were RHGS’s four quartile groups. Q1: 0.03-0.96; Q2: 0.97-1.27; Q3: 1.28-1.65; Q4: 1.66-3.90. RHGS: relative handgrip strength; HGS: handgrip strength. *p < 0.05, **p < 0.005.

**Figure 2:**
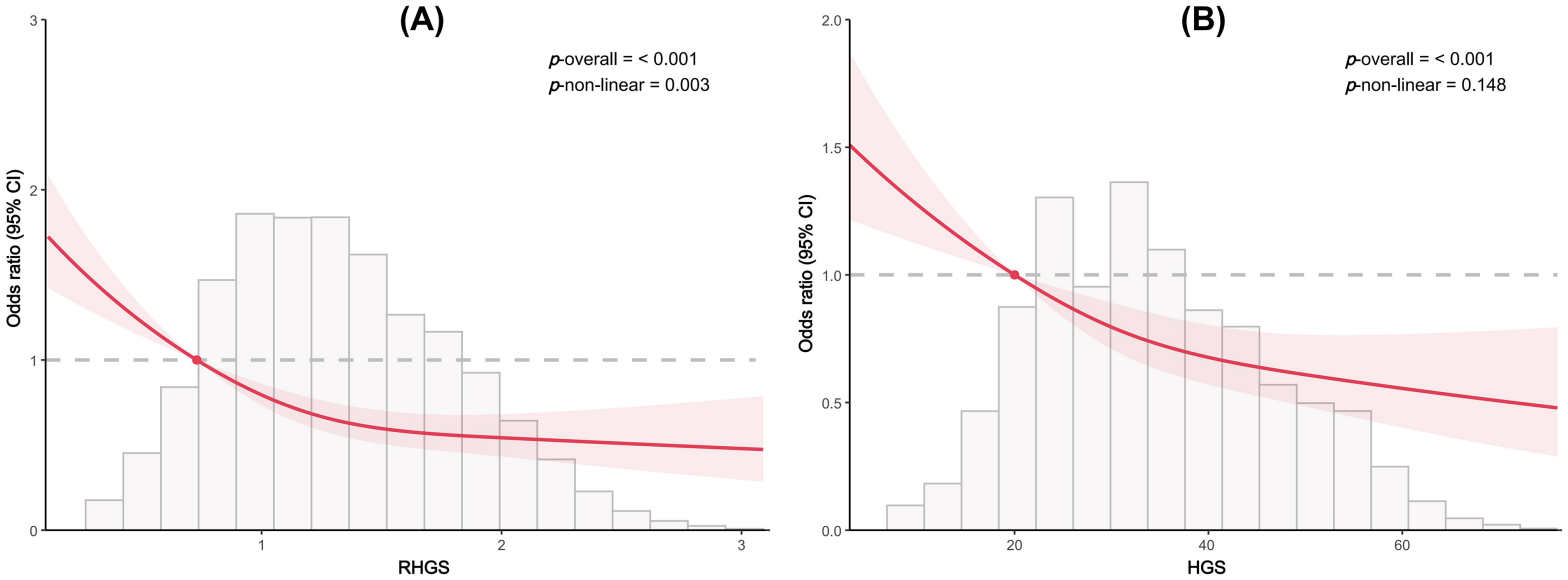
Dose-response relationship between RHGS **(A)**, HGS **(B)**, and asthma risk based on cross-sectional study.

**Figure 3:**
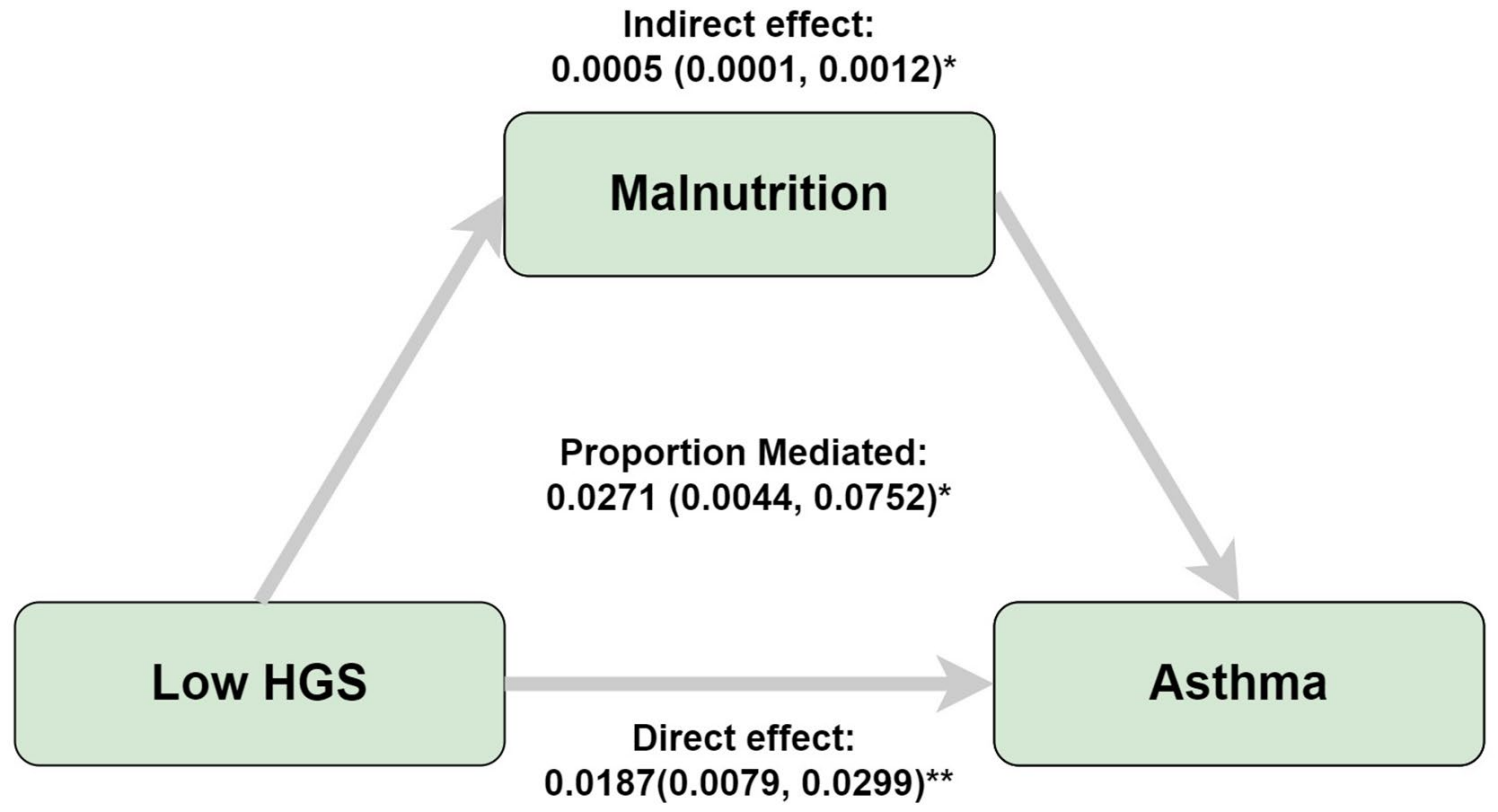
Mediation analyses of the association between low HGS and asthma risk. Adjusted by gender, country, age, education, marriage, malnutrition, smoke, drinking, heart attack, hypertension, stroke, diabetes, chronic lung disease, arthritis, cancer. *p < 0.05, **p < 0.005.

This investigation employed logistic regression analyses to quantify the relationship between handgrip status (RHGS and HGS) and asthma (Figure 1). All three logistic regression analyses demonstrated a negative correlation between RHGS and HGS and the prevalence of asthma. Individuals exhibiting low HGS had a significantly increased risk of asthma. For each unit increase in RHGS and HGS in Model Z, which accounted for all covariates, the risk of asthma decreased by 39% and 2%, respectively. Furthermore, trend tests suggested that the risk of asthma was significantly lower (*p* for trend < 0.0001) in the larger RHGS or HGS groups (Q2, Q3, Q4) than in the lowest RHGS or HGS group (Q1). According to RCS, RHGS and asthma risk had a non-linear negative association, whereas HGS had a linearly negative correlation (Figure 2). Mediation analysis was carried out to evaluate the mediating influence of nutritional status on the association between handgrip status and asthma risk. Malnutrition partially mediated the association between low HGS and asthma risk, with a mediating proportion of 2.71% (Figure 3).

### 3.3 Performance of various machine learning models in predicting asthma risk

**Figure 4:**
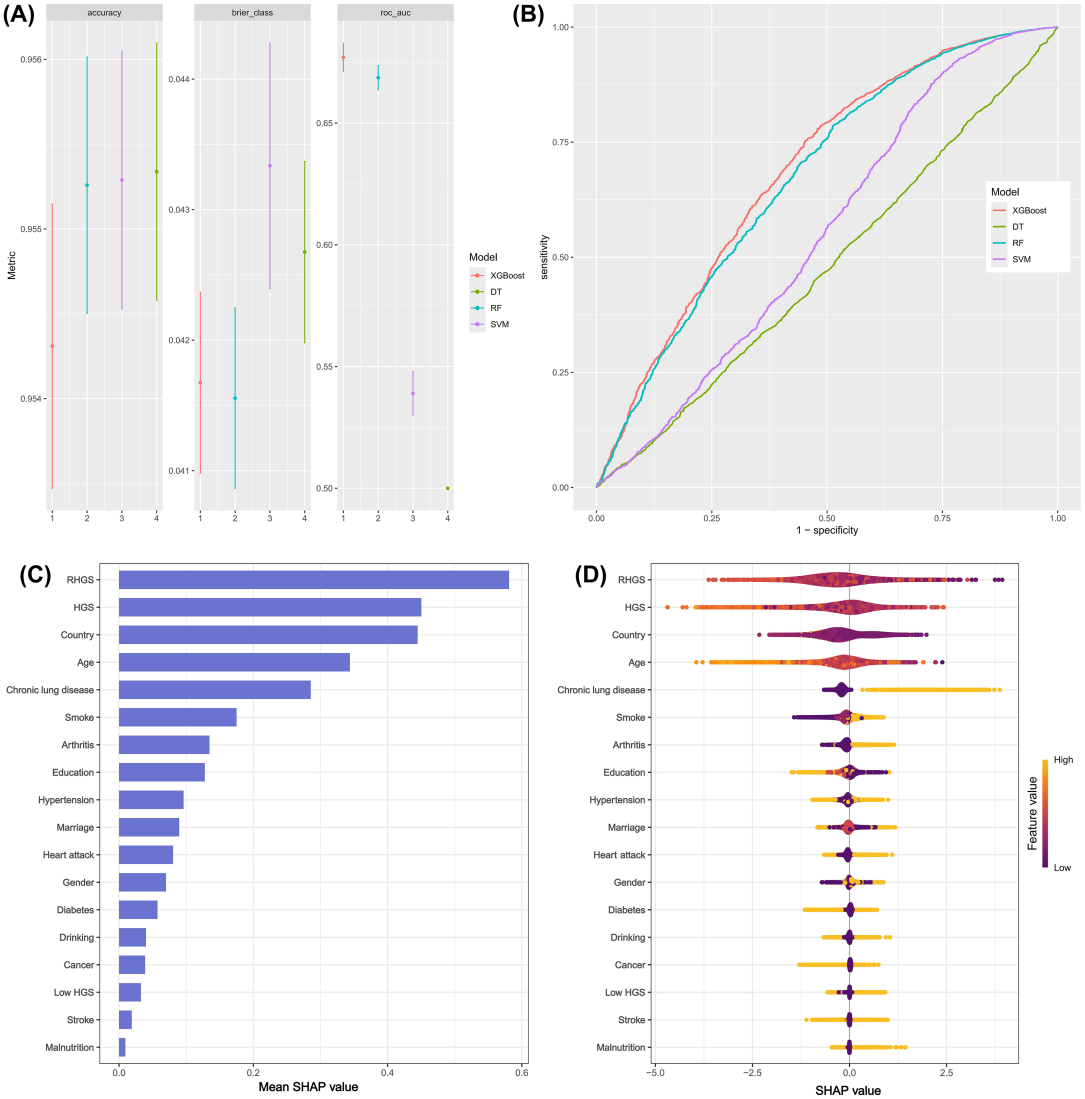
The predictive performance of different machine learning models **(A and B)**. The SHAP model provided the corresponding importance of every feature on asthma risk **(C and D)**.

This investigation developed four distinct machine learning models (SVM, RF, DT, XGBoost) to assess the performance of grip strength status in predicting asthma risk. The findings demonstrate that XGBoost outperformed the other three machine learning models, with an AUC of 0.67 (Figures 4A,B). Subsequently, the SHAP model based on XGBoost was implemented to assess the importance and positive/negative impacts of every variable on asthma risk. The SHAP model’s outcomes indicated that the top five key predictors for predicting asthma risk were RHGS, HGS, country, age, and chronic lung disease, with RHGS and HGS showing a negative correlation with asthma risk (Figures 4C,D).

### 3.4 Association between handgrip status and asthma risk based on the prospective cohort

**Figure 5:**
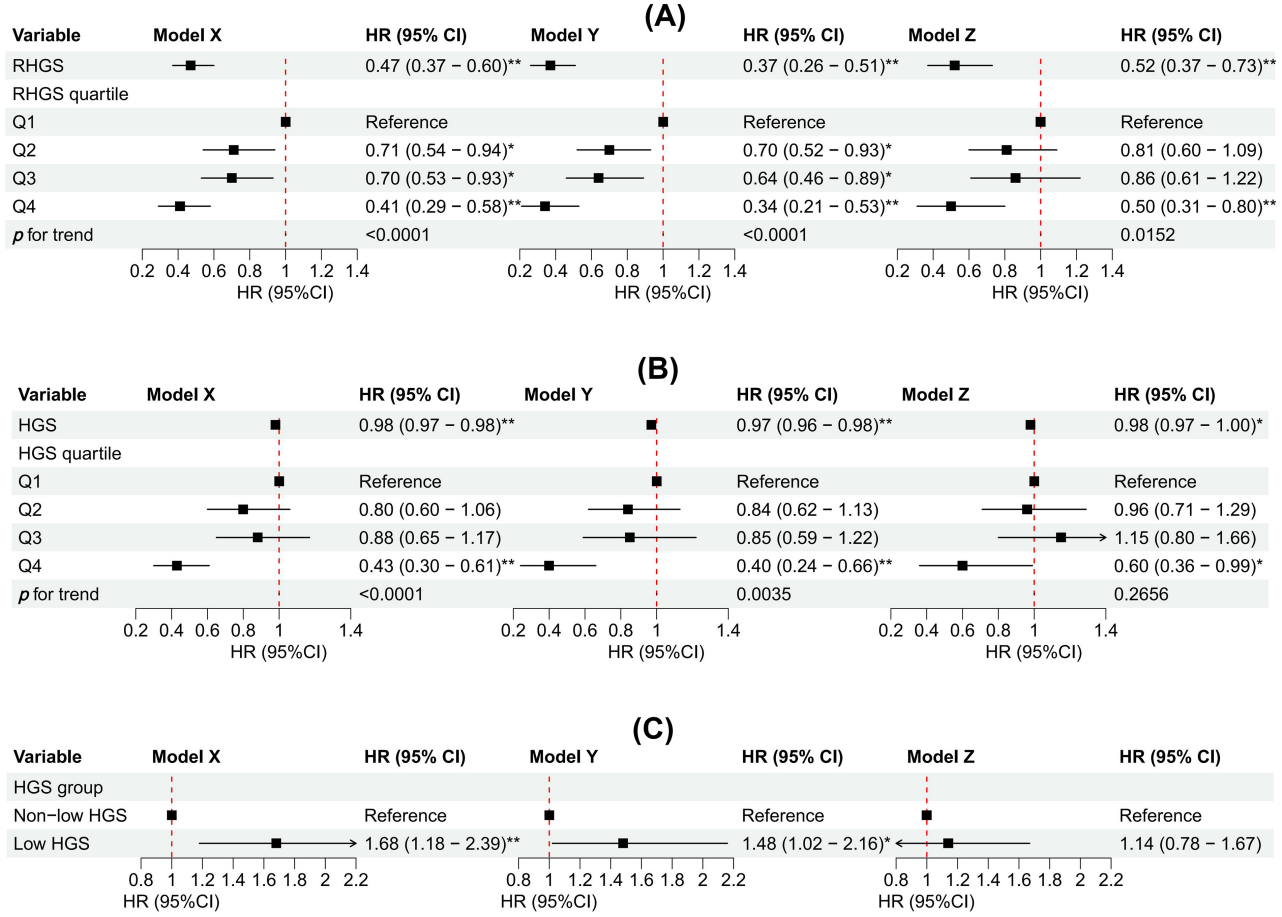
Association between RHGS **(A)**, HGS **(B)**, low HGS **(C)**, and asthma based on the prospective cohort. Model X adjusted for none. Model Y adjusted for gender, country, and age. Model Z = Model Y + adjusted for education, marriage, malnutrition, smoke, drinking, heart attack, hypertension, stroke, diabetes, chronic lung disease, arthritis, cancer. Q1-Q4 were RHGS’s four quartile groups. Q1: 0.08-0.97; Q2: 0.97-1.27; Q3: 1.28-1.65; Q4: 1.65-3.52. *p < 0.05, **p < 0.005.

**Figure 6:**
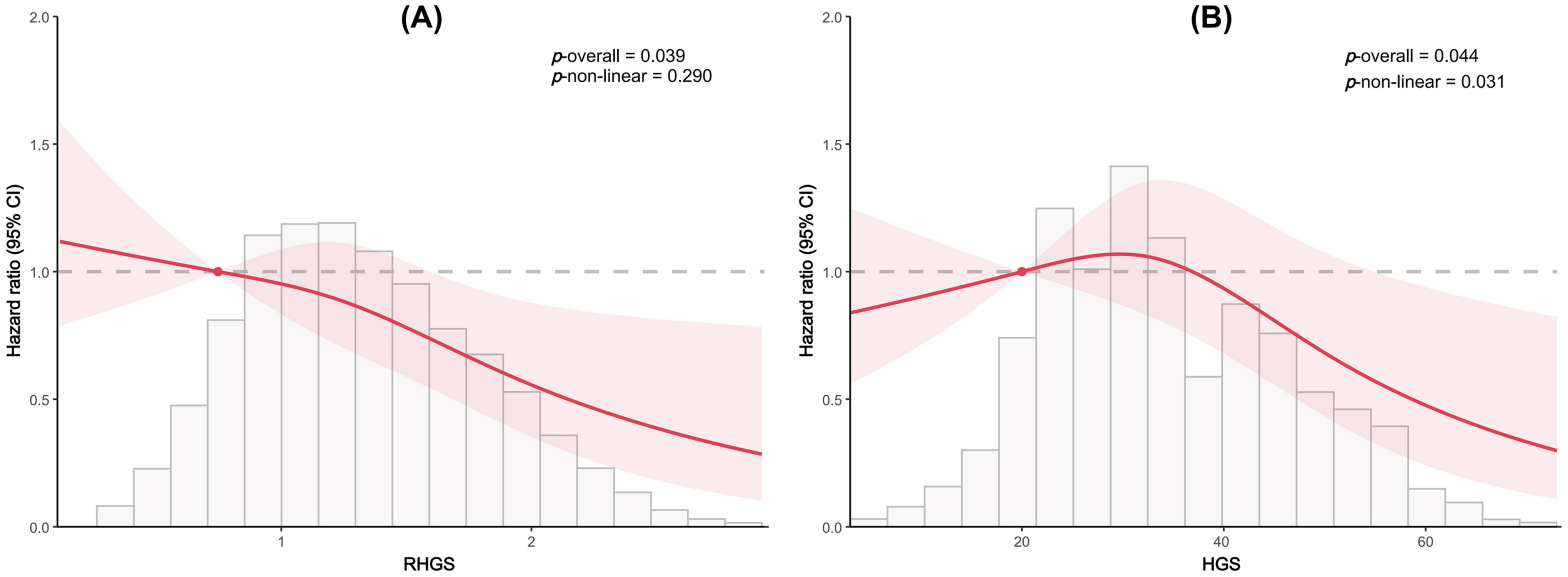
Dose-response relationship between RHGS **(A)**, HGS **(B)** and asthma risk based on the prospective cohort study.

**Figure 7:**
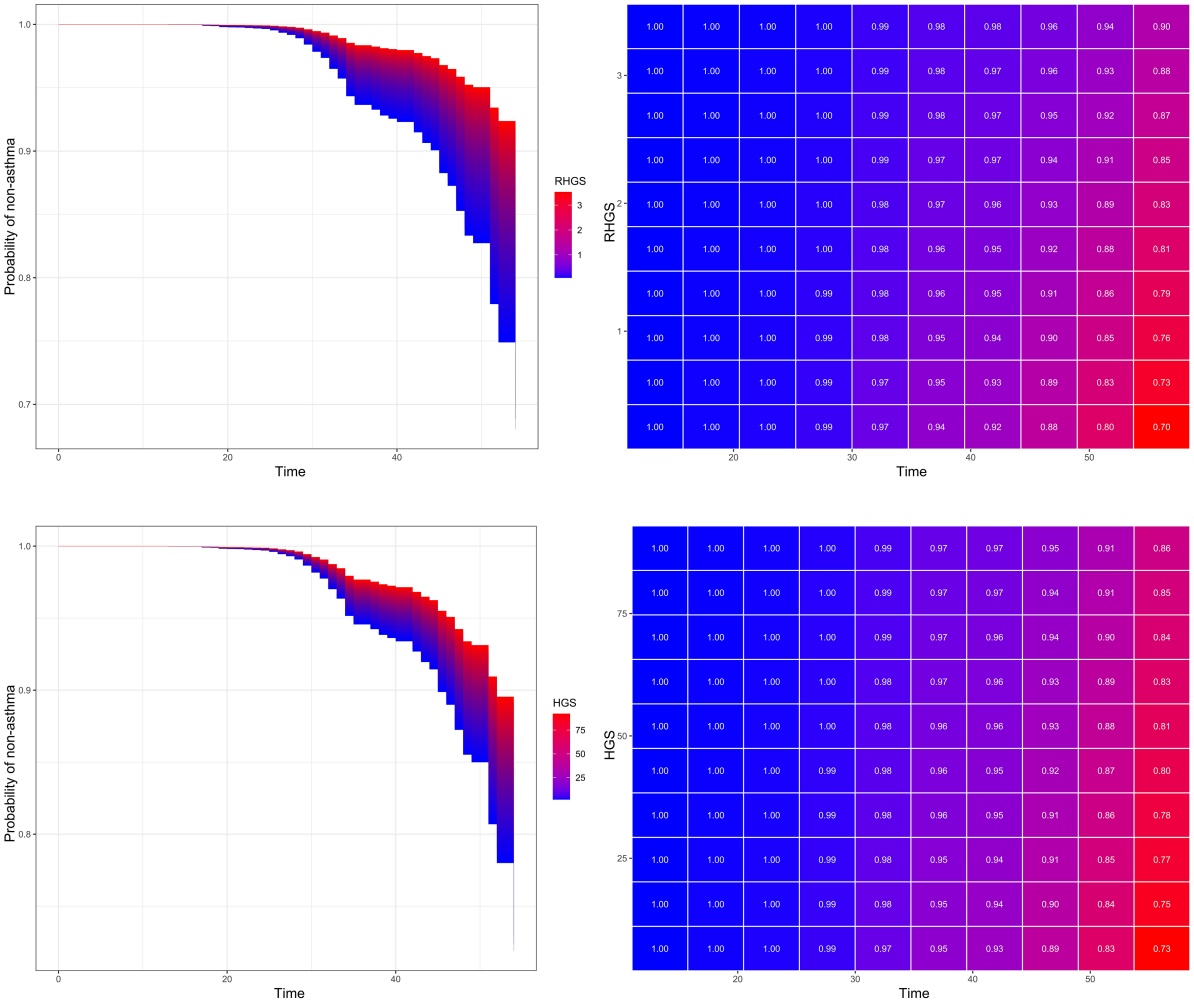
Event area plot and matrix plot displaying estimates of the handgrip status on the probability of not having asthma at the various follow-up time points, based on the Cox proportional hazards regression of model Z.

This investigation also conducted a prospective cohort study to discuss the relationship between handgrip status and asthma risk, confirming the reliability of the outcomes of the horizontal investigation. All three Cox proportional hazard models demonstrated a negative correlation between RHGS and HGS and asthma risk (Figure 5). Individuals exhibiting low HGS had a significantly increased risk of asthma. And the RCS proved that RHGS and asthma risk had a linear negative association, whereas HGS had a non-linearly negative correlation (Figure 6). The cumulative risk curve analysis revealed that the RHGS or HGS group in the lower quartile had a significantly increased risk of asthma in comparison to the higher quartile groups (Supplementary Figure S2). The findings from the event area plot and quantile plot suggested that, at the same follow-up time point, a greater RHGS or HGS corresponded with an increased likelihood of not having asthma (Figure 7).

### 3.5 Sensitivity analysis

This research also conducted several sensitivity analyses to further verify the previous results’ reliability and stability. The Supplementary material contained the results of the sensitivity analyses. The outcomes of multivariate Cox and logistic regressions, trend tests, mediation analyses, and RCS based on data after multiple imputations were consistent with previous outcomes. Both RHGS and HGS (Supplementary Tables S1, S2 and Supplementary Figure S3) were negatively correlated with asthma risk; meanwhile, malnutrition partially mediated the association between low HGS and asthma risk, with a mediation proportion of 2.29% (Supplementary Figure S4).

## 4 Discussion

This investigation suggested that both HGS and RHGS were negatively correlated with the risk of asthma, and low HGS increased the risk of asthma. Of the four machine learning models used to assess HGS and the risk of asthma, XGBoost demonstrated superior predictive performance. The SHAP model based on XGBoost suggested that the top five crucial indicators for predicting asthma risk were RHGS, HGS, country, age, and chronic lung disease. Finally, the mediation effect model suggested that malnutrition partially mediated the relationship between low HGS and asthma risk.

In clinical practice, grip strength is a simple, cost-effective test commonly used to evaluate muscular strength. Recent studies have further demonstrated that HGS not only reflects overall muscle function but also correlates with the occurrence, progression, and prognosis of various respiratory diseases. For instance, a meta-analysis by Mackenzie Holden et al. concluded that individuals with lower HGS had an elevated risk of mortality and an increased likelihood of suffering from COPD and had a lower quality of life in relation to their health (29). This association is supported by a Chinese case–control study, which found that COPD patients had significantly lower HGS compared to healthy controls, with underweight individuals (low BMI) being more prone to muscle loss (30). Similarly, research by Shah S et al. revealed that the average HGS and muscle endurance in individuals with COPD were notably lower than those of healthy populations. A significant positive correlation was observed between HGS and forced vital capacity (FVC) in men, as well as between HGS and forced expiratory volume in 1 s (FEV1) in women (31). In contrast to participants with stable COPD and those without COPD, Turan et al. (32) found that HGS was lower in those experiencing an exacerbation of their condition. Low HGS during an exacerbation may be caused by steroid usage and physical inactivity. An epidemiological study in the United States suggested that smokers with COPD typically exhibited reduced HGS, meanwhile HGS was independently linked to an increased risk of worsening the condition (33).

Additionally, several studies have explored the role of grip strength in asthma. For example, a cross-sectional study in Brazil showed that HGS was a dependable diagnostic instrument for assessing frailty risk of asthmatic individuals, proposing cutoffs of <19 for women and ≤27 for men as the appropriate thresholds for frailty in older asthmatic individuals (24). A recent prospective cohort study conducted in the UK revealed the association between HGS and various chronic respiratory diseases, which aligns with our findings that low HGS may increase asthma risk. As the decline in HGS advanced to sarcopenia, approximately 10.4% of individuals diagnosed with sarcopenia were found to have asthma (34). Su et al. (35) confirmed from a genetic perspective that HGS may serve as a potential protective factor for asthma, whereas low HGS could be considered a potential risk factor for asthma. Conversely, a study conducted in Denmark revealed that HGS correlated with FEV1 and FVC in both genders, yet it did not show any association with other asthma-related outcomes in a group primarily composed of healthy adolescents, indicating that the noted correlations may not be specific to asthma (25). In addition, a follow-up study in Finland over 22 years found no significant correlation between the magnitude of grip strength decline and the incidence of asthma (36), which differs from our study. This inconsistency may be attributed to the different follow-up periods and demographic characteristics of the populations studied. Our study’s median follow-up time of 30 months, coupled with a larger sample size, may have contributed to the clearer association we observed between grip strength and asthma risk. Nevertheless, patients with persistent asthma had a more pronounced decline in grip strength compared to those who had never had asthma throughout the follow-up period in the Finnish cohort study (36). This may be attributed to impaired lung function as a direct result of decreased muscle function, and is particularly relevant in the context of asthma, where weakened respiratory muscle strength may limit effective ventilation and exacerbate respiratory symptoms.

The findings of this research suggested that, from an epidemiological perspective, HGS may serve as a potential protective factor against asthma, while low HGS levels were associated with an increased risk of asthma, with malnutrition partially mediating this relationship. A decrease in grip strength, a surrogate for whole-body muscle strength, may reflect a synchronized weakening of respiratory muscles, suggesting impairment of lung function. Additionally, patients with muscle loss are often accompanied by a chronic low-grade inflammatory state, which may contribute to asthma development and exacerbation. Individuals with lower HGS tend to have elevated inflammatory markers, such as CRP and cytokines (37, 38), which can worsen airway inflammation and bronchial hyperresponsiveness. Furthermore, malnutrition, particularly insufficient intake of protein and energy, exacerbates muscle loss, weakening immune function and increasing systemic inflammation (39, 40), which further increases asthma risk. Our study suggests that malnutrition partially mediates the link between low HGS and asthma risk by amplifying muscle dysfunction and impairing immune responses.

In contrast to previous research, this study has the advantage of incorporating populations from multiple European countries to investigate the relationship between HGS and asthma incidence rates from both cross-sectional and longitudinal perspectives. Secondly, the efficacy of handgrip status in predicting the risk of asthma was assessed using a variety of machine learning frameworks. Finally, the mediating effect was employed to verify that the increased risk of asthma caused by low HGS was partially mediated by malnutrition. The accuracy and dependability of the research findings were further validated through a series of concurrent sensitivity analyses.

This investigation still has certain limitations. For example, the mechanism by which grip strength increases the risk of asthma is currently unclear. Qaisar et al. (41) proposed that c-terminal agrin fragment-22 (CAF22) and selected miRNAs can serve as helpful indicators for sarcopenia in asthma. Asthma diagnosis is self-reported, introducing potential recall bias or social desirability bias. The inclusion of diverse European countries introduces heterogeneity in healthcare access, dietary patterns, and cultural practices, which could influence the outcomes. Additionally, potential confounding factors, such as living environment, other chronic diseases and family medical history, may not have been considered in this study. The definition of malnutrition in this investigation is primarily based on the BMI of the study population.

## 5 Conclusion

This investigation suggested that lower HGS and RHGS were associated with a higher risk of asthma, and handgrip status serving as an independent predictive marker of asthma risk in European populations. And malnutrition partially mediated the relationship between low HGS and increased risk of asthma. Appropriate muscle exercise may help reduce the risk of asthma onset.

## Data Availability

Publicly available datasets were analyzed in this study. This data can be found: all data utilized in this research comes from the SHARE, which is openly accessible to scholars (https://releases.sharedataportal.eu).
