# Supplementary material for: Malnutrition mediates the association between handgrip status and asthma risk: an observational and prospective cohort study from multiple European countries
Source: Front Nutr. 2025 Jul 7;12:1555888. doi: 10.3389/fnut.2025.1555888 (PMC12277167; doi:10.3389/fnut.2025.1555888)
Supplement: Supplementary file 1 [file Data_Sheet_1.pdf]

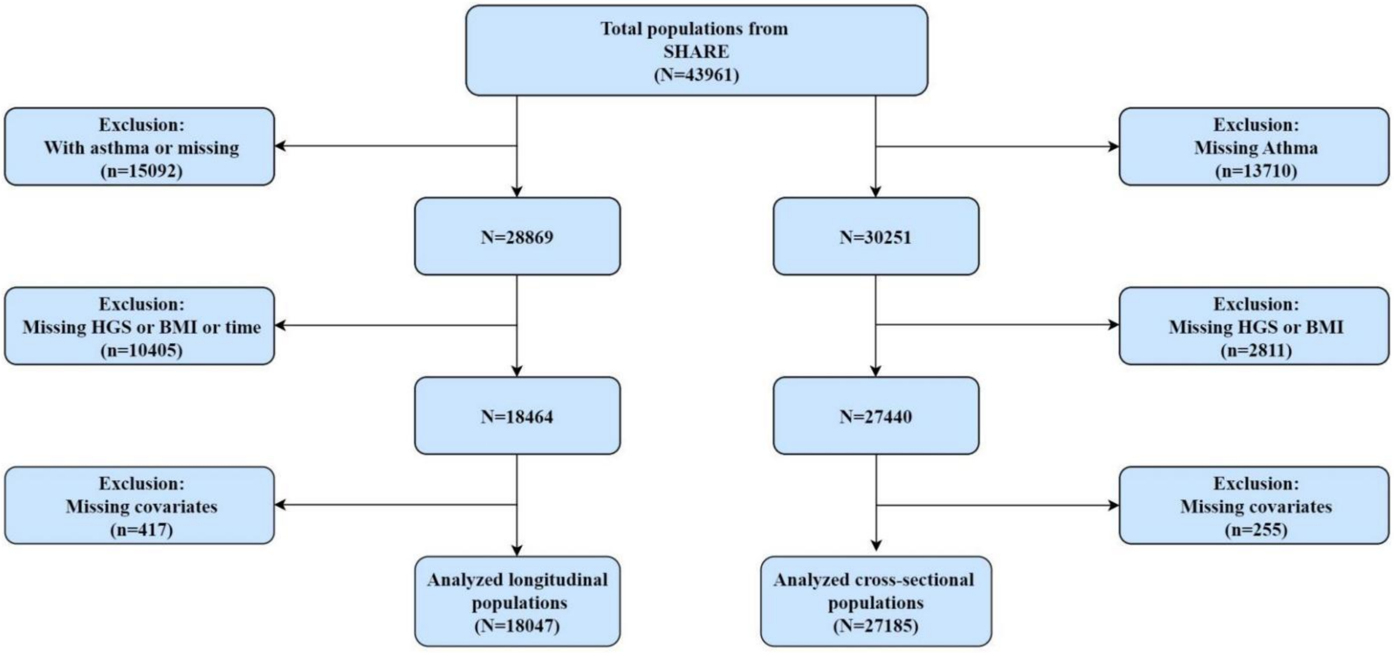

Supplementary Figure 1. Screening process diagram for cross-sectional and longitudinal populations.

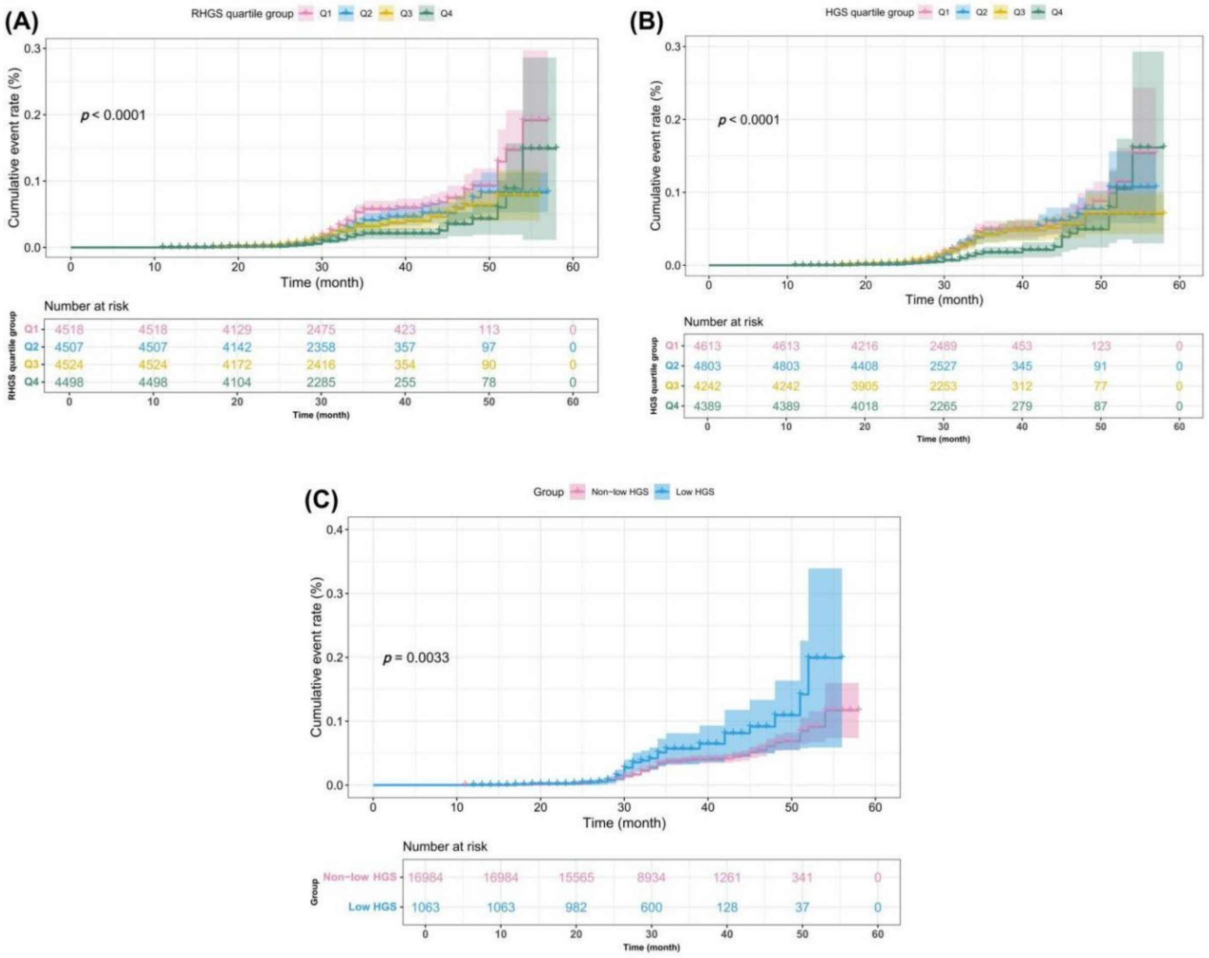

Supplementary Figure 2. Cumulative event curves of new asthma onset based on different handgrip status groups.

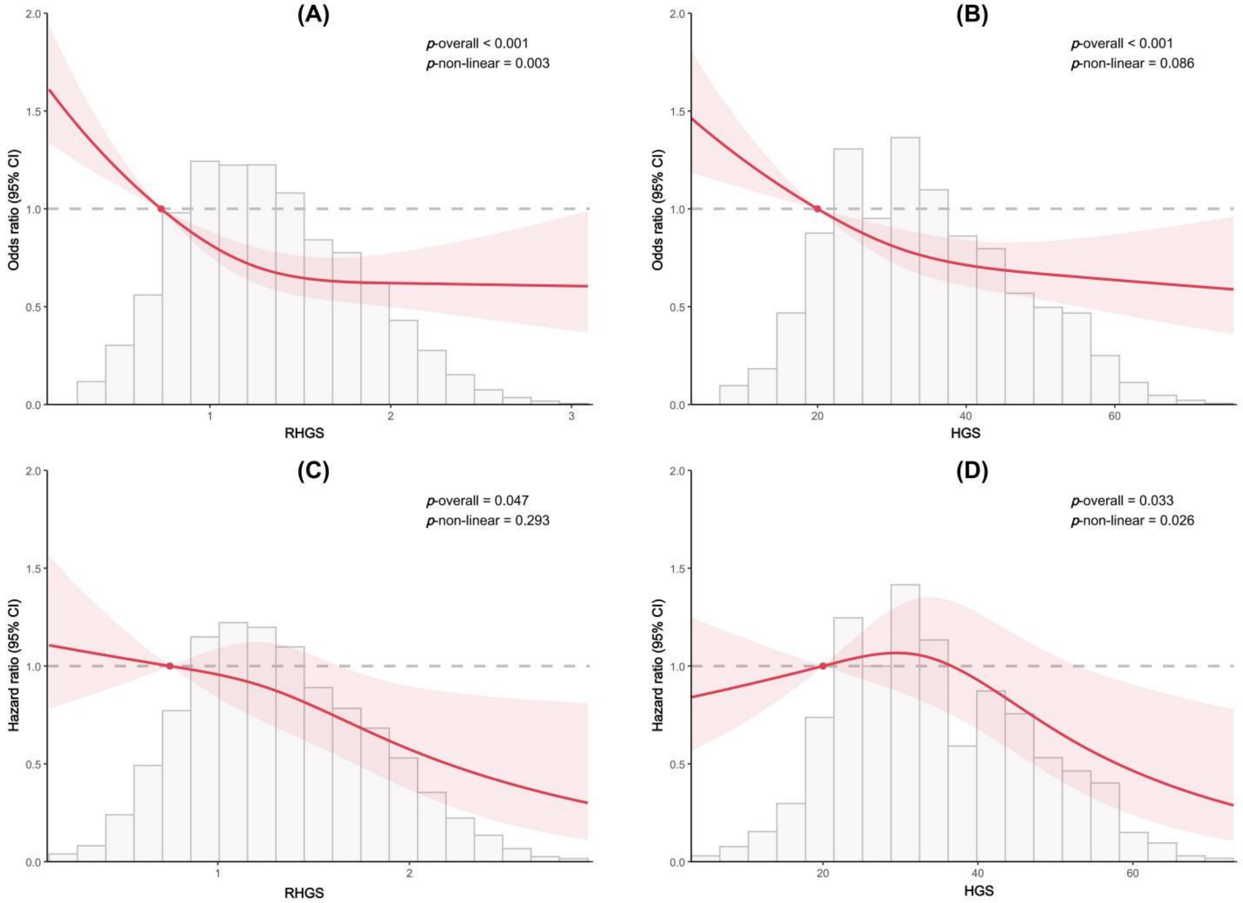

Supplementary Figure 3. Dose-response relationship between RHGS (A and C), HGS (B and D), and asthma risk based on the horizontal and longitudinal investigation of data after multiple imputations.

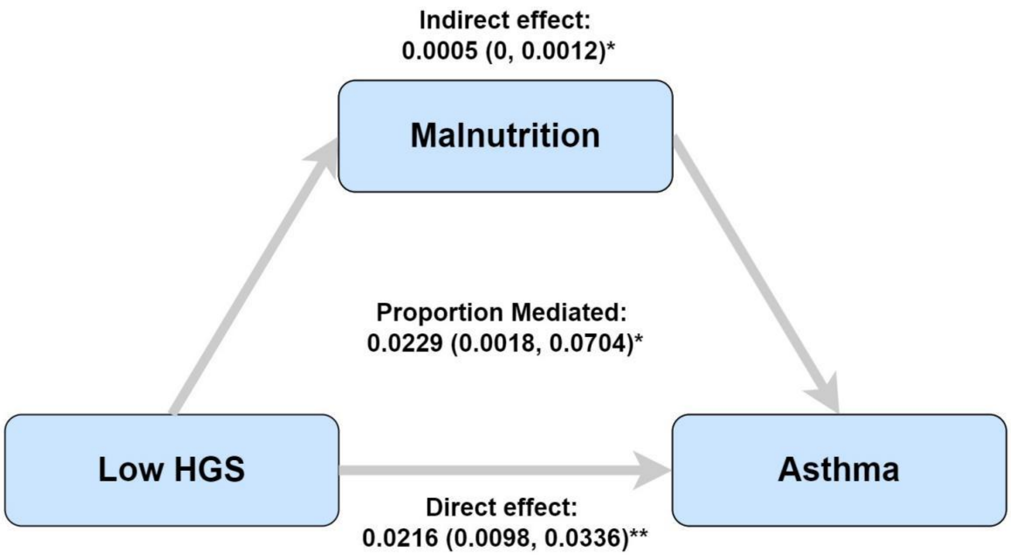

Supplementary Figure 4. Mediation analyses of the association between low HGS and asthma risk. Adjusted by gender, country, age, education, marriage, malnutrition, smoke, drinking, heart attack, hypertension, stroke, diabetes, chronic lung disease, arthritis, cancer. * $p < 0.05$, ** $p < 0.005$.

Supplementary Table 1. Association between handgrip status and asthma based on the horizontal investigation of data after multiple imputations

	**Model X**	**Model Y**	**Model Z**
	**OR (95% CI)** ***p*** **value**	**OR (95% CI)** ***p*** **value**	**OR (95% CI)** ***p*** **value**
RHGS	0.64 (0.56, 0.72) <0.0001	0.46 (0.39, 0.55) <0.0001	0.61 (0.51, 0.73) <0.0001
RHGS quartile			
Q1	Reference	Reference	Reference
Q2	0.65 (0.55, 0.75) <0.0001	0.59 (0.50, 0.70) <0.0001	0.68 (0.58, 0.81) <0.0001
Q3	0.59 (0.51, 0.70) <0.0001	0.49 (0.40, 0.59) <0.0001	0.59 (0.49, 0.72) <0.0001
Q4	0.58 (0.50, 0.69) <0.0001	0.42 (0.33, 0.52) <0.0001	0.56 (0.44, 0.70) <0.0001
p for trend	<0.0001	<0.0001	<0.0001
HGS	0.98 (0.98, 0.99) <0.0001	0.97 (0.97, 0.98) <0.0001	0.98 (0.98, 0.99) <0.0001
HGS quartile			
Q1	Reference	Reference	Reference
Q2	0.67 (0.57, 0.79) <0.0001	0.64 (0.54, 0.75) <0.0001	0.73 (0.62, 0.87) 0.0004
Q3	0.68 (0.59, 0.80) <0.0001	0.58 (0.48, 0.70) <0.0001	0.71 (0.59, 0.87) 0.0006
Q4	0.59 (0.50, 0.70) <0.0001	0.43 (0.34, 0.56) <0.0001	0.59 (0.46, 0.77) <0.0001
p for trend	<0.0001	<0.0001	<0.0001

Note: Model X adjusted for none. Model Y adjusted for gender, country, and age. Model Z = Model Y + adjusted for education, marriage, smoke, drinking, malnutrition, heart attack, hypertension, stroke, diabetes, chronic lung disease, arthritis, cancer. Q1-Q4 were RHGS's four quartile groups.

Supplementary Table 2. Association between handgrip status and asthma based on the longitudinal investigation of data after multiple imputations

	**Model X**	**Model Y**	**Model Z**
	**HR (95% CI)** ***p*** **value**	**HR (95% CI)** ***p*** **value**	**HR (95% CI)** ***p*** **value**
RHGS	0.49 (0.39, 0.63) <0.0001	0.38 (0.27, 0.53) <0.0001	0.53 (0.38, 0.75) 0.0003
RHGS quartile			
Q1	Reference	Reference	Reference
Q2	0.70 (0.53, 0.92) 0.0115	0.69 (0.51, 0.92) 0.0112	0.79 (0.58, 1.06) 0.1113
Q3	0.72 (0.55, 0.95) 0.0216	0.66 (0.48, 0.92) 0.0151	0.89 (0.63, 1.25) 0.4909
Q4	0.43 (0.31, 0.61) <0.0001	0.36 (0.23, 0.56) <0.0001	0.53 (0.33, 0.83) 0.0063
p **for trend**	<0.0001	<0.0001	0.0265
HGS	0.98 (0.97, 0.99) <0.0001	0.97 (0.96, 0.98) <0.0001	0.98 (0.97, 1.00) 0.0112
HGS quartile			
Q1	Reference	Reference	Reference
Q2	0.81 (0.61, 1.07) 0.1404	0.85 (0.63, 1.14) 0.2829	0.97 (0.72, 1.31) 0.8548
Q3	0.89 (0.67, 1.19) 0.4262	0.85 (0.59, 1.21) 0.3656	1.15 (0.80, 1.65) 0.4602
Q4	0.44 (0.31, 0.62) <0.0001	0.39 (0.24, 0.65) 0.0002	0.60 (0.36, 0.99) 0.0439
p **for trend**	<0.0001	<0.0025	0.2453

Note: Model X adjusted for none. Model Y adjusted for gender, country, and age. Model Z = Model Y + adjusted for education, marriage, smoke, drinking, malnutrition, heart attack, hypertension, stroke, diabetes, chronic lung disease, arthritis, cancer. Q1-Q4 were RHGS's four quartile groups.

Methods

1.1 Data source and study design

The Survey of Health, Ageing and Retirement in Europe (SHARE) is a multidisciplinary, longitudinal database designed to study aging populations across multiple European countries. It collects comprehensive data on the health, socioeconomic status, and social networks of individuals aged 50 and older, making it a great resource for investigating the interplay between handgrip strength, malnutrition, and asthma risk. The database’s strengths include its large sample size, cross-national coverage, and prospective design, which enable robust statistical analyses and generalizable findings (http://www.share‐project.org/organisation/share‐eric.html). In this longitudinal cohort study, we used data from the following waves: Wave 1 (baseline, 2004–2006), Wave 2 (2006–2007). The dataset includes numerous countries including Austria, Germany, Sweden, Netherlands, Spain, Italy, France, Denmark, Greece, Switzerland, Belgium, and Israel. This investigation included 27,185 participants for a cross-sectional study and 18,047 participants for a prospective cohort study from the SHARE wave 1 to 2. Screening of cross-sectional study population: (1) people without asthma (n=13710); (2) people with missing HGS or BMI (n=2811); (3) people with missing covariates (n=255). The prospective cohort study population was screened as follows: (1) individuals with asthma or missing data (n=15092); (2) individuals with missing HGS or BMI or time (n=10405); and (3) individuals with missing covariates (n=417). **Figure S1** showed the specific screening process of the population analyzed in this investigation.

1.2 Measurement of RHGS, HGS, and low HGS

The assessment of HGS was executed using a hand-held dynamometer (Smedley, S Dynamometer, TTM, Tokyo, 100 kg). The interviewer demonstrated the procedure before asking the respondent for willingness to perform the test. Medical exclusion criteria were swelling or inflammation, severe pain or recent injury, and recent surgery to the hand. Respondents were instructed to press the dynamometer with both their left and right hands, each repetition performed twice with alternations between the hands. If respondent had problem with one hand,

measurements were only taken with the other hand. The test was performed with the respondent standing upright, the upper arm parallel to the upper body and the lower arm at a 90-degree angle to the upper arm. The test could be performed also in a sitting position if necessary. Interviewers were trained for the grip strength test based on harmonized training. HGS in this investigation was defined as the highest value of either hand. RHGS (m2) = HGS (kg)/BMI (kg/m2). For the purpose of this study, a low HGS was defined based on gender-specific thresholds, following the guidelines established by the European Working Group on Sarcopenia in Older People (EWGSOP2), with a threshold of 27kg for men and 16kg for women.

1.3 Statistical analysis

For categorical data, the P-value was ascertained using the chi-square test.The Kruskal-Wallis rank-sum test was implemented to compute the P-value for continuous variables. For continuous variables that lacked a normal distribution, this research implemented the median and IQR. Categorical variables were described using proportions. Initially, this investigation employed three logistic regressions (for cross-sectional populations) and Cox proportional hazards regression analyses (based on longitudinal populations) to examine the relationship between handgrip status (RHGS, HGS, low HGS) and asthma risk. Trend tests and restrictive cubic splines (RCS), based on models that adjust for all covariates (age, country, gender, education, drinking status, smoking status, hypertension, diabetes, chronic lung disease, heart attack, stroke, arthritis, and cancer status), can further quantify the relationship between handgrip status and asthma risk. To predict asthma risk, we employed four machine learning models: Support Vector Machines (SVM), Random Forests (RF), Decision Trees (DT), and XGBoost. These models were selected for their complementary strengths in classification tasks within biomedical research. SVM was chosen for its effectiveness in high-dimensional datasets, enabling the modeling of non-linear relationships through kernel functions—relevant given the complex associations among handgrip status, malnutrition, and asthma risk. RF was included to mitigate overfitting, a key consideration with heterogeneous data from multiple European countries, and to provide feature importance metrics for identifying critical predictors. DT was selected for its interpretability, offering transparent decision paths that can inform clinical insights. XGBoost was chosen for its high predictive performance and efficiency with large datasets, aligning with the design of this

study. Each model was optimized using 5-fold cross-validation to tune hyperparameters and ensure robust performance. For SVM, we tuned the kernel type (e.g., linear, polynomial, or radial basis function) and regularization parameter via grid search. For RF, we adjusted the number of trees and maximum depth to balance accuracy and complexity. DT was tuned for maximum depth and minimum samples per leaf to preserve interpretability. XGBoost was optimized for learning rate, maximum depth, and subsample ratio using a combination of grid and random search. The area under the curve (AUC) served as the primary performance metric to identify the best model, given its ability to assess discrimination across classification thresholds in potentially imbalanced datasets. Subsequently, we applied Shapley Additive Explanations (SHAP) based on the XGBoost model—which exhibited the highest AUC among the four models—to evaluate the importance of each variable in forecasting asthma risk and to elucidate the relationships between all variables and the asthma risk. Machine models mainly used "xgboost","randomForest" ,"kernlab", "rpart", "tidymodels" package, etc. Afterwards, the cumulative risk curve was used to evaluate the relationship between handgrip status with the occurrence of asthma. This investigation also applied the area plots and matrix plots, basing on the "contsurplot" package, to evaluate the causal effect of grip strength on the absence of asthma during the follow-up period. Subsequently, to assess whether nutritional status mediates the causal association between low HGS and asthma risk, we implemented a mediation effect model (“mediation” package) using the Baron and Kenny method. This approach involved (1) regressing asthma risk on low HGS to establish the total effect, (2) regressing malnutrition on low HGS to confirm the exposure-mediator relationship, and (3) regressing asthma risk on both low HGS and malnutrition to estimate the direct and indirect effects. The mediation effect was calculated as the product of the coefficients from Steps 2 and 3, with significance tested using the Sobel test. Key assumptions included: no unmeasured confounding, no exposure-affected mediator-outcome confounders, linearity, and consistency. To validate these findings, we used bootstrapping with 1,000 resamples to generate 95% confidence intervals for the indirect effect, providing a robust, non-parametric confirmation of the mediation effect. Missing covariates included age, drinking status, education, marriage, and smoking status. The proportion of each missing covariate was less than 1%, and the missing covariates in this study were addressed through multiple imputations ("mice" package). Conduct sensitivity

analyses of the primary findings using the data generated through multiple imputations. Every statistical analysis was carried out with R 4.4.1. Statistical significance was defined as a ***p*** value of less than 0.05.
